# Radical Abdominal Trachelectomy for IB1 Cervical Cancer at 17 Weeks of Gestation: A Case Report and Literature Review

**DOI:** 10.1155/2014/926502

**Published:** 2014-12-08

**Authors:** Yoichi Aoki, Morihiko Inamine, Sugiko Ohishi, Yutaka Nagai, Hitoshi Masamoto

**Affiliations:** Department of Obstetrics and Gynecology, Graduate School of Medical Science, University of the Ryukyus, 207 Uehara, Nishihara, Okinawa 903-0215, Japan

## Abstract

*Background*. With regard to the therapy for early invasive cervical carcinoma during pregnancy, radical trachelectomy is also a treatment of choice, along with its advantages and disadvantages. *Case Report*. A 28-year-old woman, para 1-0-0-1, was diagnosed with FIGO stage IB1 squamous cell carcinoma of the cervix at 12 weeks of gestation. The patient underwent radical abdominal trachelectomy with pelvic lymphadenectomy at 17 weeks of gestation. Her pregnancy was successfully maintained after the surgery. The patient underwent a planned cesarean section at 38 weeks of gestation. A healthy baby girl weighing 2970 g was born with an Apgar score of 8/9. The mother and child in overall good health were discharged. Ten months after the delivery, there was no clinical evidence of recurrence. *Conclusions*. We believe that it is appropriate to perform radical abdominal trachelectomy in the early second trimester with preserving uterine arteries, although it is a technically challenging approach. It may be possible that radical abdominal trachelectomy during pregnancy can help women avoid the triple losses of a desired pregnancy, fertility, and motherhood.

## 1. Introduction

Cervical cancer is the most commonly diagnosed malignant tumor during pregnancy [1, 2]. The incidence of abnormal cervical cytological findings is estimated to occur in 1%–5% of all pregnancies, and the reported rate of cervical cancers ranges between 1 and 12 per 10,000 pregnancies [1–3]. The question arises regarding the choice of treatment for pregnant patients diagnosed with early invasive cervical carcinoma who wish to maintain the existing pregnancy. A few national and international recommendations or guidelines have been developed for the management of cervical cancer in pregnant women [4]. The main difficulty is the absence of a high quality evidence-based medicine to guide a consistent approach.

To date, only three possible treatment modes have been proposed with regard to the therapy for early invasive cervical carcinoma during pregnancy, which provides a possibility maintaining the pregnancy for the full term. A postponed radical surgery provides fetal maturity and therefore increased the chances of the fetus surviving outside the uterus. However, this type of treatment is still limited to patients in the late second or early third trimester with stage I cervical carcinoma. For patients with a stage IB1 tumor (≥2 cm) and negative pelvic lymph nodes, neoadjuvant chemotherapy until fetal maturity is an alternative. Several conservative management options, such as laparoscopic evaluation including lymphadenectomy or neoadjuvant chemotherapy combined with delayed therapy, have been proposed [5, 6]. Finally, radical trachelectomy is also a treatment of choice, along with its advantages and disadvantages, and is also a possible treatment option. In this study, we report the application of this technique to a patient with ongoing pregnancy and cervical cancer.

## 2. Case Report

A 28-year-old woman, para 1-0-0-1, was diagnosed with invasive squamous cell carcinoma of the cervix at 12 weeks of gestation and she was referred to the University of the Ryukyus Hospital. Specular examination showed a sessile tumor measuring approximately 2 cm in diameter on the anterior lip of the cervix. At 15 weeks of gestation, magnetic resonance imaging showed a lesion consistent with cervical carcinoma FIGO stage IB1. Both the patient and her husband were informed about the treatment options and the possible consequences of such treatments on the pregnancy. The couple was also informed about the option to perform radical abdominal trachelectomy (RAT) as a relatively new surgical procedure and all the possible consequences were discussed. The patient was told that the outcome of this procedure could not be guaranteed because not enough procedures had been globally performed to yield reliable conclusions. The patient wished to maintain the pregnancy, and both parents signed a written consent agreeing to RAT. The institutional review board of our university approved this treatment.

The patient underwent RAT with pelvic lymphadenectomy under general anesthesia in August 2013 at 17 weeks of gestation. To prevent uterine contraction, 50 mg of indometacin sodium rectal suppository was administered on the morning of the surgery; 25 mg of indometacin sodium rectal suppository was immediately administered after surgery and every 6 h thereafter (4 times in total). In addition, 250 mg of 17-alpha-hydroxyprogesterone caproate was intramuscularly administered 60 min immediately before the surgery and administered once a week thereafter until 36 weeks of gestation. An enlarged pregnant uterus prevented the operative field; therefore it was secured by manually putting aside the uterus because indomethacin sodium and sevoflurane were effective enough for the softening of the uterine muscle.

**Figure 1:**
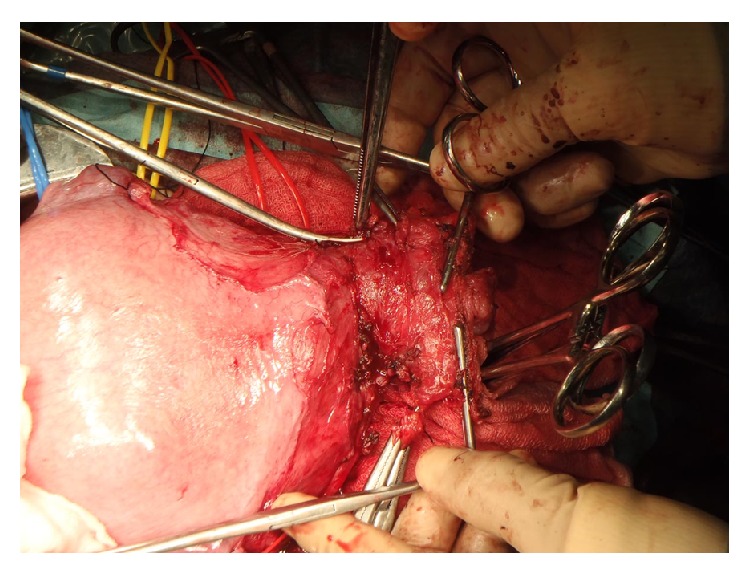
The vaginal wall was clamped with forceps and cut off, with preserving bilateral uterine arteries.

**Figure 2:**
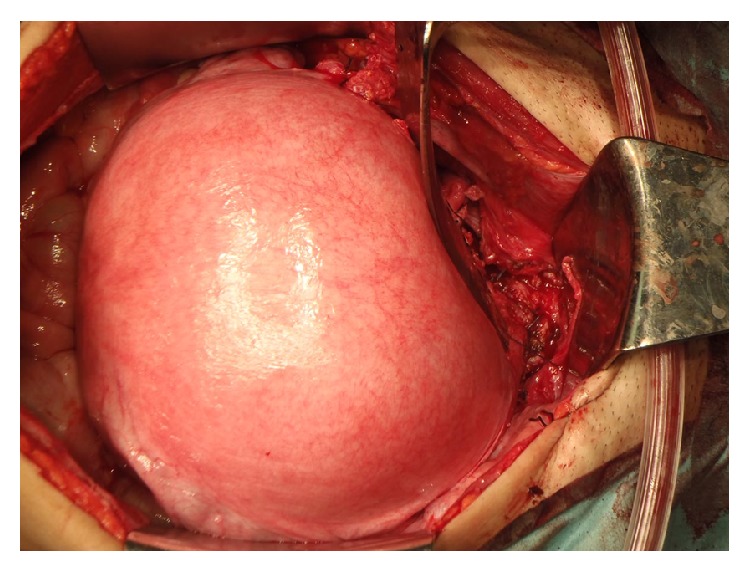
The vaginal wall and the remaining uterine cervix were anastomosed after applying a nonabsorbable cerclage suture to the lowest possible site of the uterine corpus.

In our case, pelvic lymphadenectomy was performed. The frozen section of the lymph nodes, bilateral obturator, and external iliac nodes revealed no metastases. By dissociating ureters from the retroperitoneum, bilateral uterine arteries were identified and preserved, and subsequently cardinal, uterosacral, and rectovaginal ligaments were transected. After processing the anterior and posterior vesicouterine ligaments, the vagina was cut off (Figure 1) and the cervix was transected below the isthmus of the uterus. The sample was sent for frozen-section analysis, showing the endocervical margin was negative for cancer. A nonabsorbable cerclage suture was applied to the lowest possible site of the uterine corpus with a hydroxybutyrate-coated polyester suture, taking care not to rupture any amniotic membranes. The vaginal wall and the remaining uterine cervix were anastomosed (Figure 2). The excised specimen included 2.8 cm of uterine cervix with 1 cm of vaginal cuff and 3 cm of each parametrium. A cytological smear from the remaining cervix was negative. An intraoperative ultrasound examination of the fetus, amniotic fluid, fetal heart rate, and placental site was performed. The surgery took 6 h 23 min, resulting in a blood loss of 2510 mL. Eight units of MAP were transfused. There were no intraoperative or immediate complications after the surgery. The final pathology on the trachelectomy specimen revealed stromal invasion less than one-third with no apparent lymphovascular and parametrial involvement; there were negative margins and 13 negative lymph nodes. After the surgery, a cervical smear was performed at 8-week intervals. No abnormalities were observed and her pregnancy was successfully maintained.

Antenatal ultrasound was performed monthly, showing normal structural images, normal fetal biometry, and an adequate cervical length (≥20 mm). The patient underwent a planned cesarean section at 38 weeks of gestation. A healthy baby girl weighing 2970 g was born with an Apgar score of 8/9. The pH in the umbilical artery was 7.282. The mother and child in overall good health were discharged on day 15 after surgery. Ten months after the delivery, a cervical smear test revealed normal findings with regular colposcopic findings. There was no clinical evidence of recurrence.

## 3. Discussion

Radical trachelectomy is used for women who are not pregnant with early-stage disease and cervical lesions measuring ≤2 cm without nodal involvement and who wanted to preserve their fertility. The procedure consists of a pelvic lymphadenectomy followed by the removal of the cervix together with the surrounding parametria with preservation of the uterine corpus and ovaries. So far, 20 cases of women with cervical cancer, including our case (4 stage IA2, 15 IB1, and one IB2) who underwent radical trachelectomy during pregnancy, have been reported [4]. Of these, ten trachelectomies were done via the abdominal route and 10 via the vaginal route. The approach to radical trachelectomy is abdominal and vaginal, with all their advantages and disadvantages.

In terms of RAT (Table 1), ten patients were operated on between 7 and 22 weeks of gestation [1, 7–10]. No patient had nodal spread at the time of RAT. All patients were free of disease during follow-up of 8–54 months. Apart from the technically challenging procedure, RAT during pregnancy was associated with significant blood loss (200–2510 mL) and prolonged surgery (3.5–7.5 h). Furthermore, the rate of fetal loss after this procedure was high (4 fetal losses occurred 0–16 days after surgery). Three of the four patients who underwent RAT earlier than 14 weeks of gestation had miscarriages. Two cases treated with RAT at 7 weeks and 8 weeks of gestation had an abortion one day after the surgery, and one woman treated with RAT at 13 weeks of gestation suffered a late abortion on day 17 after surgery. This suggests that fetuses in the first trimester may not be able to tolerate the RAT procedures. The RAT treatment at 22 weeks of gestation in another patient resulted in intrauterine fetal death 4 h after the surgery [9]. A sufficiently wide operative field may not have been obtained because of the enlarged gravid uterus in this case. In addition, they ligated bilateral uterine arteries during trachelectomy. They stressed that the major concern was the altered blood supply of the uterus because the only remaining uterine blood supply in these patients was the ovarian vessels and these vessels seemed to adapt to the needs of pregnancy if they took over the blood supply of uterus in early gestational weeks. Actually, although the fetal autopsy revealed no abnormal findings, the placenta showed hypoxic changes without any sign of abruption. This case suggested that their flow might be insufficient in late gestation, resulting in loss of the pregnancy. The uterine arteries should be preserved as much as possible when performing the RAT during pregnancy.

These obstetrical results suggest a correlation between a high early abortion rate and RAT. Therefore, on the basis of these data, a radical trachelectomy during pregnancy is not recommended by the guidelines of a second international consensus meeting for gynecologic cancers in pregnancy [4]. However, the other five women, including our case, who underwent surgery between 15 weeks and 19 weeks of gestation, gave birth to healthy live babies [1, 7, 8, 10]. We believe that it is appropriate to perform RAT in the early second trimester with preserving uterine arteries. Detailed investigation of similar cases would reveal the efficacy and safety of this relatively new procedure, although it is a technically challenging approach and the importance of experience and centralization should be emphasized. It may be possible that RAT during pregnancy can help women avoid the triple losses of a desired pregnancy, fertility, and motherhood.

## Figures and Tables

**Table 1:** Obstetrical and oncological outcome of radical abdominal trachelectomy during pregnancy reported in the literature.

Number	Stage	Histology	Radical abdominal trachelectomy outcome					Pregnancy outcome	Cancer prognosis	Author, year
			GA at surgery	Uterine artery	Number of removed LNs	Operation time (h)	Blood loss (mL)			
1	IB1	SCC	7 w	Preserved	NA	NA	NA	Abortion (7 w)	NED (median 40 months, range, 10–54 months)	Ung$\overset{´}{\text{a}}$r et al., 2006 [7]
2	IB1	SCC	8 w	Preserved	NA	NA	NA	Abortion (8 w)		
3	IB1	SCC	9 w	Preserved	NA	NA	NA	C/S (38 w), 3220 g, a girl		
4	IB1	SCC	13 w	Preserved	NA	NA	NA	Abortion (15 w)	Number 1–5	Number 1–5
5	IA2	SCC	18 w	Preserved	NA	NA	NA	C/S (39 w), 2980 g, a boy		

6	IB1	SCC	19 w	NA	NA	5	450	C/S, PROM (36 w), 2580 g, a boy	NED (16 months)	Mandic et al., 2009 [1]
7	IB1	Lymphoepithelial	15 w	Lt. ligated	9	3.5	1600	C/S (39 w)	NA	Abu-Rustum et al., 2010 [8]
8	IB2	SCC	22 w	Bil. ligated	NA	NA	200	IUFD (22 w), Placenta: hypoxic change	NA	Karateke et al., 2010 [9]
9	IB1	SCC	15 w	Rt. ligated	16	7.5	1200	C/S (37 w), 2584 g, a girl	NED (12 months)	Enomoto et al., 2011 [10]
10	IB1	SCC	17 w	Preserved	13	6.3	2510	C/S (38 w), 2970 g, a girl	NED (10 months)	Present case

GA: gestational age; SCC: squamous cell carcinoma; C/S: cesarean section; PROM: premature rupture of membrane; NED: no evidence of disease; NA: not addressed; LNs: lymph nodes.

## References

[1] Mandic A., Novakovic P., Nincic D., Zivaljevic M., Rajovic J. (2009). Radical abdominal trachelectomy in the 19th gestation week in patients with early invasive cervical carcinoma: case study and overview of literature. *The American Journal of Obstetrics and Gynecology*.

[2] Takushi M., Moromizato H., Sakumoto K., Kanazawa K. (2002). Management of invasive carcinoma of the uterine cervix associated with pregnancy: outcome of intentional delay in treatment. *Gynecologic Oncology*.

[3] Hunter M. I., Tewari K., Monk B. J. (2008). Cervical neoplasia in pregnancy. Part 2. Current treatment of invasive disease. *The American Journal of Obstetrics and Gynecology*.

[4] Amant F., Halaska M. J., Fumagalli M., Steffensen K. D., Lok C., Van Calsteren K., Han S. N., Mir O., Fruscio R., Uzan C., Maxwell C., Dekrem J., Strauven G., Gziri M. M., Kesic V., Berveiller P., Van Den Heuvel F., Ottevanger P. B., Vergote I., Lishner M., Morice P., Nulman I. (2014). Gynecologic cancers in pregnancy: guidelines of a second international consensus meeting. *International Journal of Gynecological Cancer*.

[5] Alouini S., Rida K., Mathevet P. (2008). Cervical cancer complicating pregnancy: implications of laparoscopic lymphadenectomy. *Gynecologic Oncology*.

[6] Palaia I., Pernice M., Graziano M., Bellati F., Panici P. B. (2007). Neoadjuvant chemotherapy plus radical surgery in locally advanced cervical cancer during pregnancy: a case report. *American Journal of Obstetrics and Gynecology*.

[7] Ungár L., Smith J. R., Pálfalvi L., del Priore G. (2006). Abdominal radical trachelectomy during pregnancy to preserve pregnancy and fertility. *Obstetrics and Gynecology*.

[8] Abu-Rustum N. R., Tal M. N., DeLair D., Shih K., Sonoda Y. (2010). Radical abdominal trachelectomy for stage IB1 cervical cancer at 15-week gestation. *Gynecologic Oncology*.

[9] Karateke A., Cam C., Celik C., Baykal B., Tug N., Ozbasli E., Tosun O. A. (2010). Radical trachelectomy in late pregnancy: Is it an option?. *European Journal of Obstetrics Gynecology and Reproductive Biology*.

[10] Enomoto T., Yoshino K., Fujita M., Miyoshi Y., Ueda Y., Koyama S., Kimura T., Tomimatsu T. (2011). A successful case of abdominal radical trachelectomy for cervical cancer during pregnancy. *European Journal of Obstetrics & Gynecology and Reproductive Biology*.

